# Microbial Community Succession and Its Association with Characteristic Flavor Substances During Natural Fermentation of Pickled Pepper

**DOI:** 10.3390/microorganisms14102303

**Published:** 2026-10-10

**Authors:** Ling Zhu, Weirong Xu, Yisheng Tang, Yaping Hu, Dan Qin, Lijun Ou, Zhixu Zhang

**Affiliations:** 1 College of Horticulture, Hunan Agricultural University, Changsha 410128, China; 2 Yuelushan Laboratory, Changsha 410128, China; 3 State Key Laboratory of Subhealth Intervention Technology, Changsha 410128, China; 4 College of Food Science and Technology, Hunan Agricultural University, Changsha 410128, China

**Keywords:** pickled pepper, natural fermentation, characteristic flavor substances, microbial community succession, correlation analysis

## Abstract

Pickled pepper is a traditional product which has been favored by consumers. The richness of its flavor is mainly determined by the complexity and diversity of microorganisms. By analyzing the succession law of the microbial community in NFPP (Naturally fermented Pickled Pepper) within 18 days’ natural fermentation of GCP (Green rice Chili Pepper) and BCP (Beauty Chili Pepper), the experiment elucidated the variation trend of the characteristic flavor substances and explored eight dominant bacteria and seven dominant fungi in GCP and seven dominant bacteria and five dominant fungi in BCP which are flavor-related. Further research found that fermentation stages can be divided into an early stage (0–7 d), middle stage (8–13 d) and late stage (13–18 d). The characteristic flavor is formed step by step and flavor-associated microbes were classified.

## 1. Introduction

Naturally Fermented Pickled Peppers (NFPP) are foods that develop typical fermented flavors through the action of microorganisms and endogenous enzymes under natural conditions [1]. Since the fermentation process of pickled peppers mostly relies on dominant strains in the natural state to achieve product fermentation, the microbial species are diverse, the community structure is complex, and the metabolic pathways are various [2]. The characteristic flavors of pickled peppers mainly come from non-volatile compounds and volatile compounds. Non-volatile compounds mainly include organic acids, free amino acids, reducing sugars, polypeptides, capsaicin, etc., which endow the product with various tastes such as sweet, sour, bitter, salty, umami, and spicy; volatile compounds mainly include alcohols, aldehydes, acids, ketones, hydrocarbons, esters, isothiocyanates, and phenols, etc., which give pickled peppers their aroma [3,4,5]. The important microorganisms related to flavor formation are also dominant strains with strong correlations to flavor substances. Studies have shown that bacteria are the main microorganisms in the fermentation process of pickled peppers, and the dominant bacterial group is lactic acid bacteria [6]. The initial microorganisms from the surface of raw materials utilize the nutrients in the raw materials for growth and metabolism. As lactic acid bacteria multiply, the lactic acid and acetic acid produced by metabolism increase the total acid in the environment, and microorganisms with poor tolerance are gradually eliminated, forming a later fermentation pattern dominated by acid-tolerant lactic acid bacteria [7]. The enzymes produced by lactic acid bacteria during fermentation hydrolyze macromolecular food components into flavor precursors and convert them into aroma compounds. Therefore, lactic acid bacteria are the main microorganisms responsible for the flavor formation of pickled peppers. The three key metabolic pathways of lactic acid bacteria involved in the production of volatile compounds are carbohydrate metabolism, proteolysis and amino acid catabolism, and lipid decomposition and fatty acid metabolism. The most important metabolic function is to ferment carbohydrates to produce lactic acid, organic acids, polyols, etc., providing precursor substances for the transformation of flavor substances [8,9]. Currently, there are relatively few studies on the microbial community succession patterns during the natural fermentation of pickled peppers. Analyzing the correlation between fermentation microorganisms and the flavor characteristics of pickled peppers is of great significance for a deeper understanding of the flavor formation mechanism of microorganisms. This paper selected two varieties commonly used in pickled pepper processing, namely green rice peppers and beauty peppers from Yunnan, as raw materials to explore the changes in characteristic flavor substances and microbial community succession patterns during the fermentation of pickled peppers. By analyzing the changes in characteristic flavor substances at different fermentation periods of NFPP, the main microbial species affecting the changes in characteristic aroma substances during the fermentation process of NFPP were determined, revealing the mechanism by which microorganisms affect the changes in characteristic flavor substances of pickled peppers, providing a theoretical basis and technical support for the flavor improvement and quality control of NFPP [1,10].

## 2. Materials and Methods

### 2.1. Experimental Materials

Two chili varieties were picked from the plant base of Honghe Hopen Food Co. LTD. of Jianshui County in Honghe Prefecture, Yunnan, China. The chilies were grown simultaneously and picked on 21 July 2023. *Capsicum annuum* L. is 5–8 cm in length and 1.2–1.5 cm in diameter; *Capsicum annuum var. conoides* is 7–12 cm in length and 0.8–1.0 cm in diameter. Both were in the best harvesting period for practical production, and 2 kg of each sample was processed to pickled chili pepper. One weighed about 3 kg and was kept at −40 °C for later use.

### 2.2. Processing of NFPP

Two chili varieties were selected for fermentation using traditional NFPP technology without spicy ingredients to compare differences in the fermented products. The main processing flow was as follows [11]:

Procedure 1—Picking and cleaning: Fresh pepper samples were uncapped and washed for 10 min, and then water was filtered out.

Procedure 2—Drying in the air: The washed pepper samples were placed in a ventilated place to naturally dry for 12 h.

Procedure 3—Fermentation: 4% NaCl was added to the chili samples and then placed in a 1 L jar and sealed for fermentation at 30 °C for 18 days.

Procedure 4—Pasteurization: The peppers were bottled and pasteurized at 85 °C for 20 min and then cooled at room temperature (about 25 °C) for later use.

The two chili varieties were processed using the above procedure and bottled in 27 jars. The fermented sample of each chili variety was selected at 0, 1, 3, 7, 13, 15, 18 fermentation days. Each sample was taken from 3 different jars and mixed evenly, grouped as numbered L1 to L7 for *Capsicum annuum* L. (common name: Green Chili Pepper, GCP) and M1 to M7 for *Capsicum annuum var. conoides* (common name: Beauty Chili Pepper, BCP).

### 2.3. Methods

#### 2.3.1. Lactic Acid

Lactic acid was measured using the modified method according to GB 5009.157-2016 [12] National Health and Family Planning Commission of the People’s Republic of China & State Administration for Market Regulation, 2016). First, 1.50 g of the crushed pickled pepper sample (accurate to 0.01 g) was weighed and put into a 50 mL centrifuge tube. Then, 20 mL water was added and homogenized for 2 min under 10,000 rpm. The mixture was centrifuged under 4000 rpm for 5 min, and the upper layer of the extract was transferred into a 50 mL volumetric flask. The residue was re-extracted with 20 mL water, and the extract was combined and metered in the same volumetric flask. The sample was filtered through a 0.45 μm membrane for analysis. A standard solution of lactic acid was prepared at 25,000 μg/mL, diluted with 0.1% phosphoric acid solution at ratios of 25, 50, 125, 250, 500 times, respectively, to draw a standard curve.

HPLC conditions: Agilent C18 column (4.6 mm × 250 mm, 5 μm); eluent: 0.1% phosphoric acid solution (A)/methanol (B) = 97.5:2.5 (*v*/*v*); flow rate:1 mL/min; wave number, 210 nm; column temperature, 40 °C; sample size, 20 µL.

#### 2.3.2. Capsaicin

Capsaicin was measured using the method from GB 21226-2007 ([13] National Health and Family Planning Commission of the People’s Republic of China & State Administration for Market Regulation, 2007). First, 12.50 g chili pepper was selected and dried at 50 °C for 2 h; a 1 g sample was weighed and mixed with 25 mL methyl-tetrahydrofuran in a flask. The flask was put under a 60 °C water bath in ultrasonic surroundings for 30 min. The steps were repeated twice, and the extract was combined for later use.

Accurately weighing 0.01 g capsaicin and 0.0001 g dihydrocapsaicin was used to draw a standard curve. A 10 µL sample was filtered using 20 µm filter membrane and injected in the Agilent HPLC instruments to measure the content.

HPLC Conditions: Agilent C18 column (4.6 mm × 250 mm, 5 μm); eluent: CH_3_OH (A)/water (B) = 75:25; flow rate:1 mL/min; wave number, 280 nm; column temperature, 30 °C; sample size, 10 µL.

#### 2.3.3. Free Amino Acids ([14] National Health and Family Planning Commission of the People’s Republic of China & State Administration for Market Regulation, 2020)

A 0.1000 g freeze-dried sample was accurately weighed and placed into 50 mL centrifuge tubes. Then, 20 mL ultrapure water was added and extracted ultrasonically 30 min (250 W, 450 kHz), and the sample was centrifuged at 8000 r/min for 15 min to get the supernatant. The process was repeated 3 times and metered in a 100 mL volumetric flask and filtered with a 0.22 μm membrane for later use.

HPLC Conditions: Waters AccQ. TagTM (3.9 mm × 150 mm), Part No. WAT052885; eluent: AccQ. TagTM (A)/acetonitrile (B) = 40:60; flow rate: 1 mL/min; wave number, 248 nm; column temperature, 37 °C; sample size, 10 µL.

Derivation of samples and standards: The standard sample was diluted 10 times with 0.1 mol/L hydrochloric acid; a 10 μL diluted standard sample was mixed with 20 μL A solution, vortex mixed evenly and 70 μL Buffer was added, vortex mixed and placed in 55 °C oven for 10 min.

#### 2.3.4. Volatile Compounds [2]

A 2.5 g pickled pepper sample was accurately weighed and placed into a 15 mL headspace sampling bottle; 3 mL saturated NaCl solution and 2-undecanol (8 μL, 0.16 mg/mL) were added, respectively, vortexed 30 s and placed in a 70 °C heating block to equilibrate for 15 min to allow the volatile components of the pickled pepper to fully volatilize. An SPME needle was inserted into the extraction vial, maintaining the headspace adsorption at 70 °C for 40 min. Subsequently, the extraction head was quickly inserted into the GC inlet and the fiber tip was extracted with desorption at 250 °C for 5 min.

GC–O–MS was performed with an Agilent 7890B GC system, which was fitted with a standard split/splitless inlet, coupled to an Agilent 5977A mass selective detector, and equipped with a Gerstel MPS2 XL multipurpose sampler, a Gerstel cryotrap system, and a Gerstel olfactory detection port 3. The instruments were controlled using Agilent Mass Hunter data acquisition software (version B.07.00) in conjunction with Gerstel Maestro software (version 1.4.8.14). The pretreatment conditions and column temperature used for GC–O analysis were as follows: DB-5MS (30 m × 250 μm, 0.25 μm), the carrier gas: He (99.999%); flow rate: 1 mL/min, non-flowing, injection port temperature: 250 °C. The temperature ramping conditions were 40 °C for 3 min, then rising at 5 °C/min to 150 °C, and then rising at 10 °C/min to 250 °C for 5 min. The ion source temperature was 230 °C, the electron energy was 70 eV, and the mass scan range was *m*/*z* 33–500. OAV: The mean concentration of each compound was calculated using the following formula: concentration of an odorant = (concentration of internal standard × peak area of compound)/peak area of internal standard. The identification of compounds was performed using the NIST11 mass spectrum database at a quality match score of greater than 85%, the retention index (RI), and the aroma characteristics perceived through sniffing. The RI of each compound was calculated using a series of n-alkanes (C8–C40) (Vandendool & Kratz, 1963). The odor threshold of each odorant was determined with reference to the compilation of Odour Threshold published by Oliemans Punter & Partners BV, The Netherlands. After quantifying each volatile compound, the odor threshold of each volatile substance in water was queried and the odor activity value was calculated according to the following formula: OAV = C*_i_*/OT*_i_* (C*_i_*: Actual concentration of compound *i*; OT_i_: Odor threshold concentration of compound *i* in water).

#### 2.3.5. Total Bacteria and Fungi Count [15] National Health and Family Planning Commission of the People’s Republic of China & State Administration for Market Regulation, 2022; [16] National Health and Family Planning Commission of the People’s Republic of China & State Administration for Market Regulation, 2016)

The plate count method is adopted as reference GB 4789.2-2022 & GB 4789.15-2016.

#### 2.3.6. Metagenomic Sequencing

Microbial genomic DNA from different samples was extracted using the E.Z.N.A.^®^ Stool DNA Kit (Omega Bio-tek, Inc., Norcross, GA 30071, USA). The targeted amplification regions and corresponding primers were as follows: the 16S V4 region amplified with primer pair 515F/806R was used to assess bacterial diversity; the 18S V4 region amplified with primer pair 528F/706R was applied to characterize eukaryotic microbial diversity; and the ITS1 region amplified with primer pair ITS5-1737F/ITS2-2043R was adopted for fungal diversity identification. In addition, alternative amplified regions contained 16S V3-V4, 16S V4-V5 and 16S V5-V7 for bacteria; archaeal 16S V4-V5 and 16S V8; as well as 18S V9 and ITS2 regions. All PCR reactions were performed in a total volume containing 15 µL of Phusion^®^ High-Fidelity PCR Master Mix, 0.2 µM primer, and 10 ng genomic DNA template. Thermal cycling was carried out as follows: an initial denaturation at 98 °C for 1 min; 30 cycles of denaturation at 98 °C for 10 s, annealing at 50 °C for 30 s, and extension at 72 °C for 30 s; and a final extension at 72 °C for 5 min. The PCR amplicons were first verified by electrophoresis on a 2% (*w*/*v*) agarose gel. Products of the expected size were subsequently purified using magnetic beads and quantified with a microplate reader. Based on the measured concentrations, individual PCR products were pooled in equimolar amounts to construct the sequencing library. The pooled DNA library was prepared following the standard Illumina protocol, which included end-repair, A-tailing, sequencing-adapter ligation, and purification. The concentration and quality of the final library were determined using a Qubit fluorometer and quantitative PCR (Q-PCR), respectively. Libraries meeting the quality criteria were sequenced on the Illumina MiSeq platform at Novogene (Beijing, China) using a paired-end 250 bp (PE250) sequencing strategy. The dataset was deposited in the Novo Magic database under Project X101SC23101687-Z01.

### 2.4. Data Analysis

Statistical analysis was performed using SPSS Statistics 26 (IBM, Armonk, NY, USA). All experiments were done in triplicate, and the data were assessed for normality using the Shapiro–Wilk test and analyzed using the t-test or Mann–Whitney U/Wilcoxon signed-rank test, as appropriate. Correlation analysis between species of microbes and characteristic flavor substances were performed using Spearman’s rank correlation coefficient. Pictures were drawn with Origin Pro 2022 (OriginLab Corporation, Northapton, MA, USA).

## 3. Results and Discussion

### 3.1. Variation of Flavor Compounds in NFPP

#### 3.1.1. Variation of Acids in NFPP

**Figure 1:**
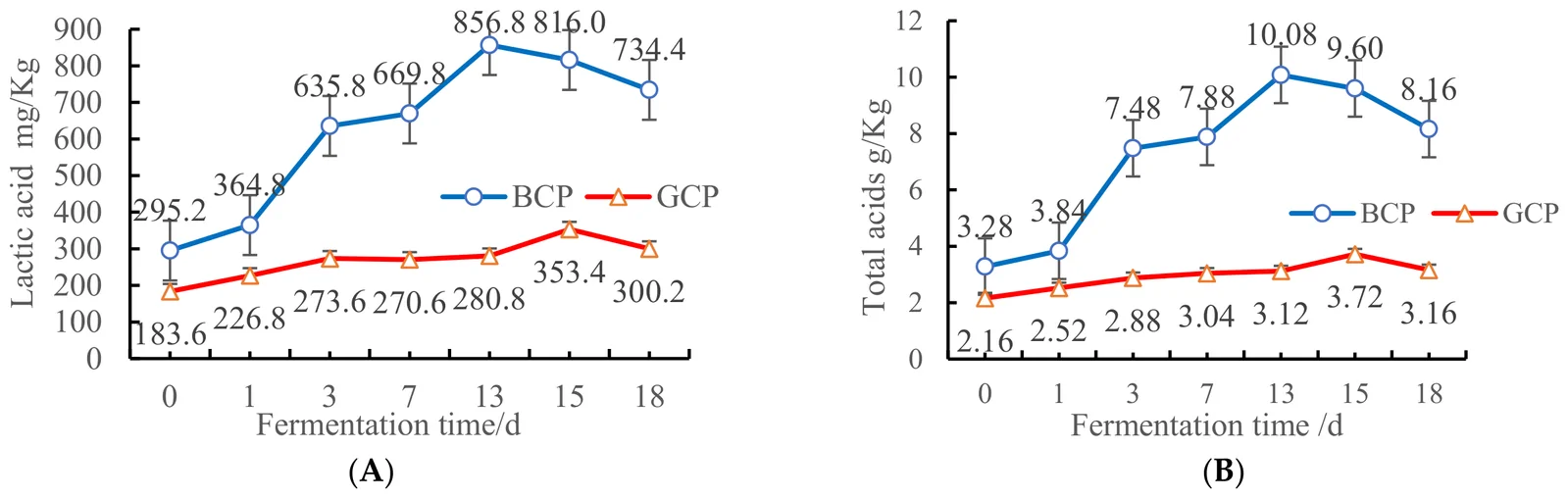
Variation of lactic acid (**A**) and total acids (**B**) at different fermentation times in NFPP processed from BCP and GCP.

Acidic substances are the most crucial flavor compounds which formed core flavor of NFPP. Lactic acid accounts for approximately 10% of the total acid content, serving as a key characteristic flavor substance. It can be observed from Figure 1, both total acid and lactic acid content follow an identical trend of rising first and declining later. Both substances increase rapidly in the early fermentation stage, and their subsequent decrease signals the termination of fermentation. Lactic acid bacteria act as the predominant microbial group that produces lactic acid, and they are also the dominant bacteria throughout NFPP fermentation. While producing lactic acid, the bacteria also hydrolyze proteins into polypeptides and free amino acids, which contribute to the distinctive lactic acid flavor. NFPP fermented from GCP yield lower acid production, which may be attributed to the higher content of capsaicinoids inhibiting the growth of lactic acid bacteria [17].

#### 3.1.2. Variation of Capsaicin and Dihydrocapsaicin in NFPP

**Figure 2:**
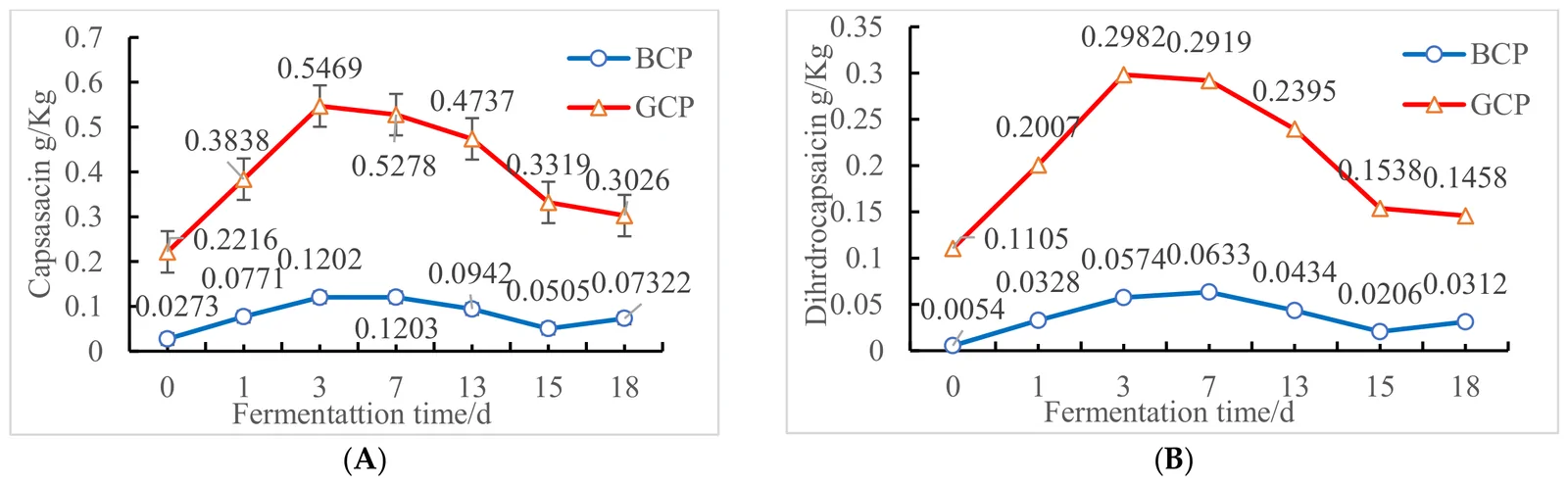
Variation of capsaicin (**A**) and dihydrocapsaicin (**B**) at different fermentation times in NFPP processed from BCP and GCP.

Capsaicin is the primary substance responsible for the spiciness of NFPP. As shown in Figure 2, the capsaicin content of both types rises initially and then falls. The peak content of capsaicinoids appears on the third day of fermentation, followed by a gradual decrease. The capsaicin content of GCP is more than ten times that of BCP, exerting a notable influence on the flavor formation of NFPP.

#### 3.1.3. Variation of Volatile Flavor Substances in NFPP

**Figure A1:**
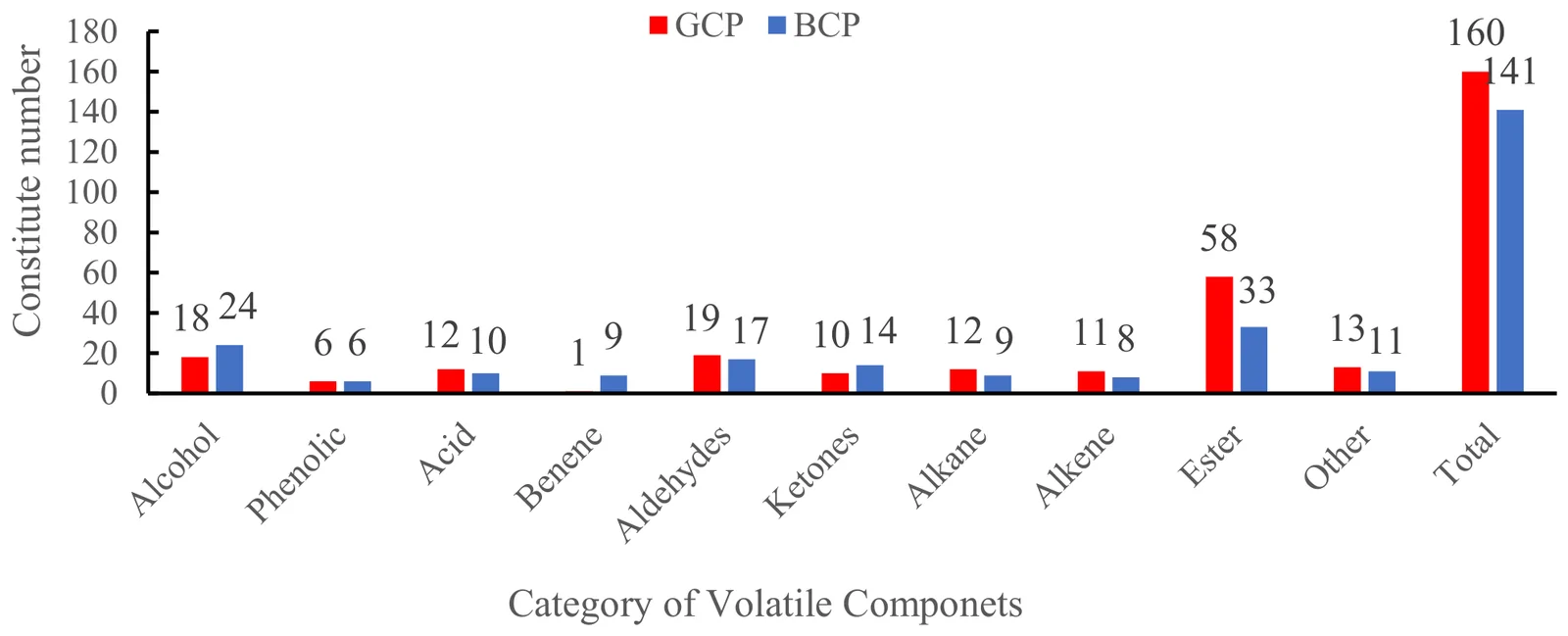
The number of volatile components of GCP anda BCP.

**Figure A2:**
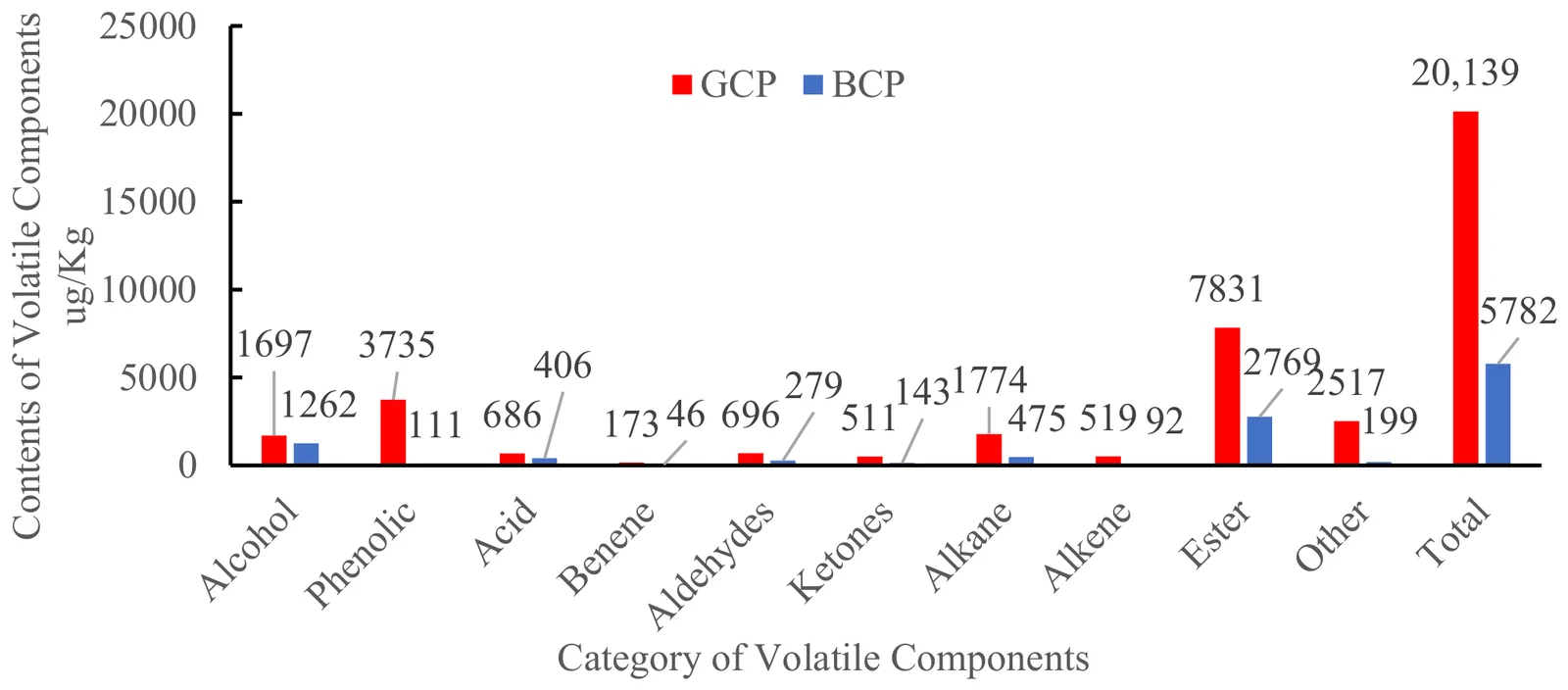
The content of volatile components in GCP and BCP.

During the natural fermentation of NFPP, the content of volatile flavor substances varies significantly across different fermentation periods. A total of 160 volatile flavor substances were detected in GCP and 142 in BCP via GC-MS. As illustrated in Appendix Figure A1 and Figure A2, esters constituted the most abundant category of volatile components in both NFPP, followed by aldehydes and alcohols, indicating a high compositional similarity between the two varieties. Total content of volatile components in GCP reached 20,139 µg/Kg, which is considerably higher than 5782 µg/Kg of BCP.

#### 3.1.4. Variation of Free Amino Acids in NFPP [18]

Free amino acids in pickled peppers are mainly generated from protein hydrolysis mediated by microorganisms and enzymes during fermentation, and different amino acid types endow NFPP with distinct flavor attributes.

A total of 10 non-essential amino acids and 6 essential amino acids were produced throughout fermentation. The total free amino acid content of GCP gradually increased and stabilized in the late fermentation stage, with a maximum value of 3226 mg/Kg. By contrast, the total free amino acids of BCP presented a continuous declining trend and tended to be steady in the mid-fermentation period, hitting a minimum value of 2204 mg/Kg. (See Appendix Table A1 and Table A2).

### 3.2. Variation of Bacterial and Fungal Populations During the Fermentation of NFPP

**Figure 3:**
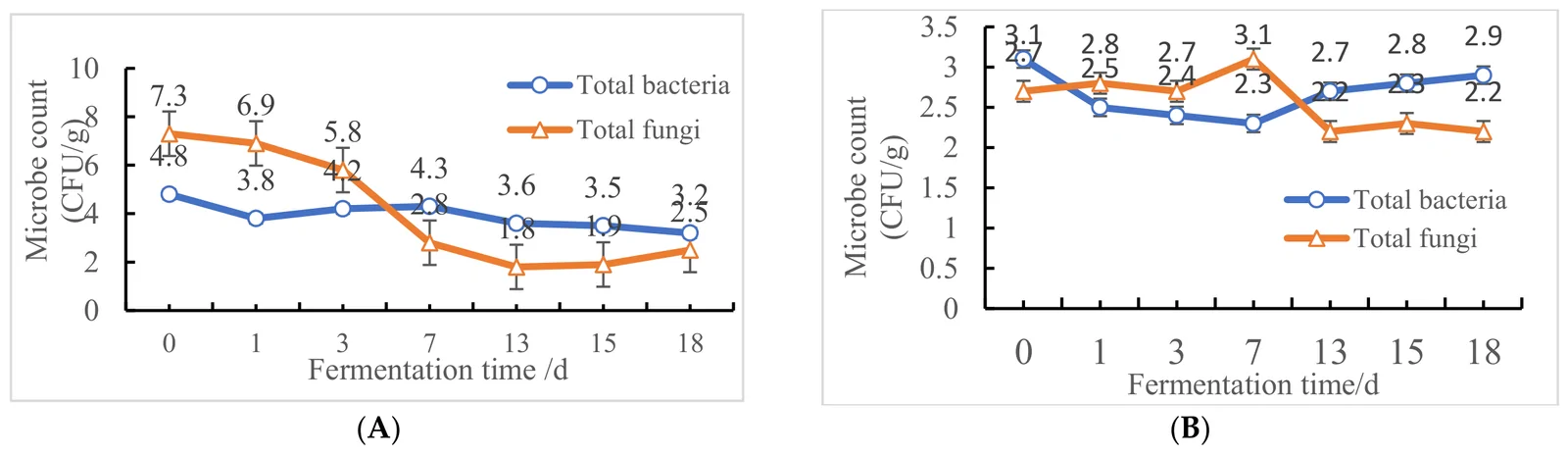
Variation of the bacteria and fungi at different fermentation times in NFPP processed from GCP (**A**) and BCP (**B**).

The predominant microorganisms in naturally fermented pickled peppers are bacteria and fungi. As shown in Figure 3, the fungal population exhibited an overall declining trend and was predominant in the early stages of fermentation. In contrast, the bacterial population remained relatively stable and became dominant in the later stages of fermentation. A notably more pronounced decrease in the fungal population was observed during the fermentation of green chili peppers (A), which is possibly attributable to their relatively higher capsaicin content exerting inhibitory effect on fungi [19].

### 3.3. Succession Pattern of Microbial Communities During the Fermentation of NFPP

#### 3.3.1. Analysis of Sequencing Data Results

A minimum of 61,346 high-quality sequences was obtained in this sequencing effort, with effective sequences constituting over 95% of the total, thereby meeting the sequencing requirements. The number of bacterial OTUs in green chili peppers decreased by 27, while the number of fungal OTUs showed a more substantial reduction of 302. This indicates a declining trend in the directly observable number of microbial species during the natural fermentation of green chili peppers, with a more pronounced change in fungal diversity.

#### 3.3.2. Beta Diversity Analysis of Microbial Communities

**Figure 4:**
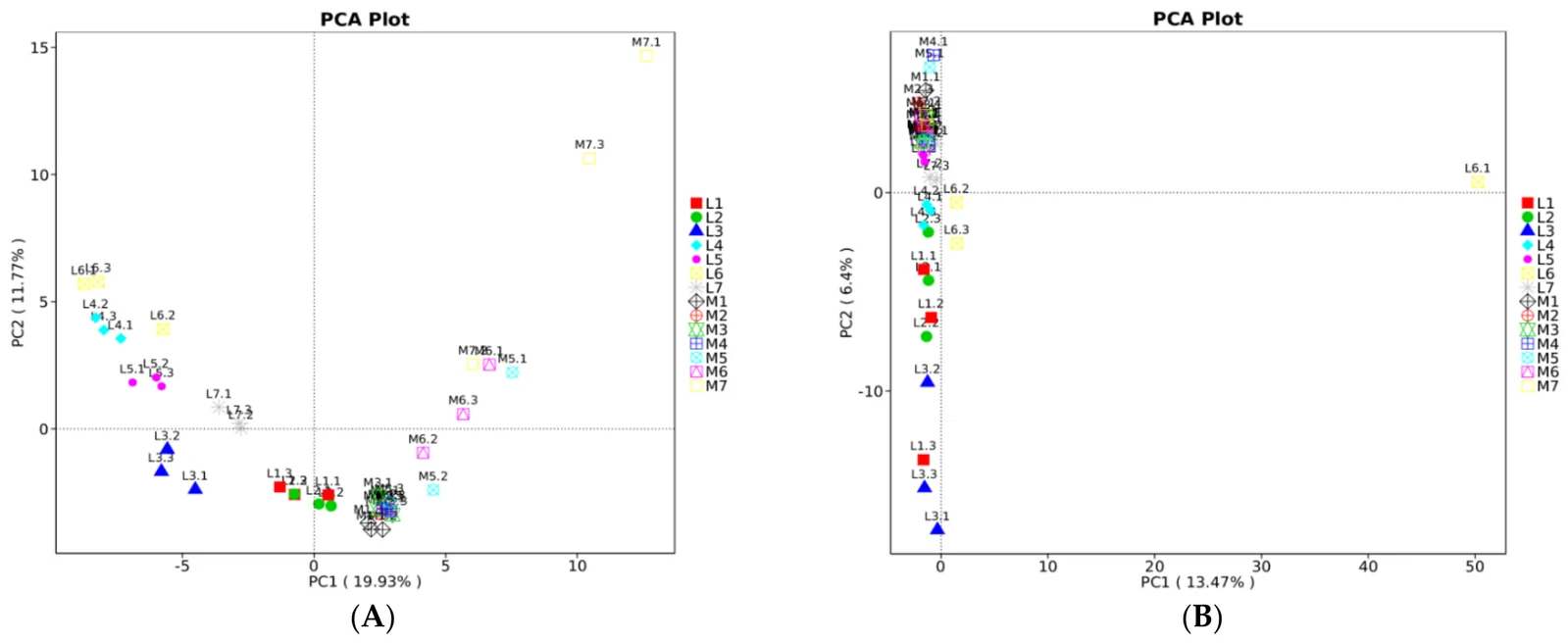
PCA analysis of species diversity of bacterial (**A**) fungal (**B**) community at different fermentation times in NFPP processed from GCP (L1-L7 represent 0, 1, 3, 7, 13, 15, 18 days) and BCP (M1-M7 represent 0, 1, 3, 7, 13, 15, 18 days).

As shown in Figure 4A, samples L1 and L2 are clustered near the boundary of the third and fourth quadrants, indicating similar bacterial communities on 0 and 1st fermentation days in GCP. L3, located in the third quadrant, is distant from other components, reflecting a distinct community structure on the 3rd day. L4–L7 are all situated in the second quadrant with short inter-distances, showing stable communities during the mid-to-late fermentation stages of GCP. M1–M4 are tightly clustered in the fourth quadrant, suggesting minimal change in the early fermentation of BCP. M5 and M6, placed in the first and fourth quadrants respectively, remain close, indicating continued stability in mid-to-late stages. M7 in the first quadrant shows dispersed replicates, possibly due to variation captured by multiple principal components.

Overall, the two types of peppers occupy separate regions in the ordination plot, and the separation tends to increase with fermentation time, implying that differences in bacterial community structures widen progressively, likely driven by certain microbiota in each variety [20].

As illustrated in Figure 4B, L1–L4 are distributed in the third quadrant with considerable distances, indicating changes in the fungal community structure during early to middle stages of fermentation in GCP. L5 and L7 are located in the second quadrant, while L6 is in the fourth quadrant. L5–L7 are relatively close to each other, suggesting that the fungal community structure stabilizes during the later stages of fermentation in GCP. From M1 to M7, the samples are largely concentrated in a confined area within the second quadrant, indicating that the fungal community structure remains relatively consistent during the fermentation in BCP. The distances between the sample groups of the two types of pepper gradually decrease on the PCA plot as fermentation progresses, suggesting that the fungal community structures become similar during the mid to late stages of fermentation. This phenomenon may be attributed to the continuous acid production by microorganisms during fermentation, which gradually acidifies the fermentation environment. Since most fungi are less acid-tolerant than bacteria, the diversity of the fungal community tends to decrease in the later stages of fermentation.

#### 3.3.3. UPGMA Cluster Analysis of Microbial Communities

**Figure 5:**
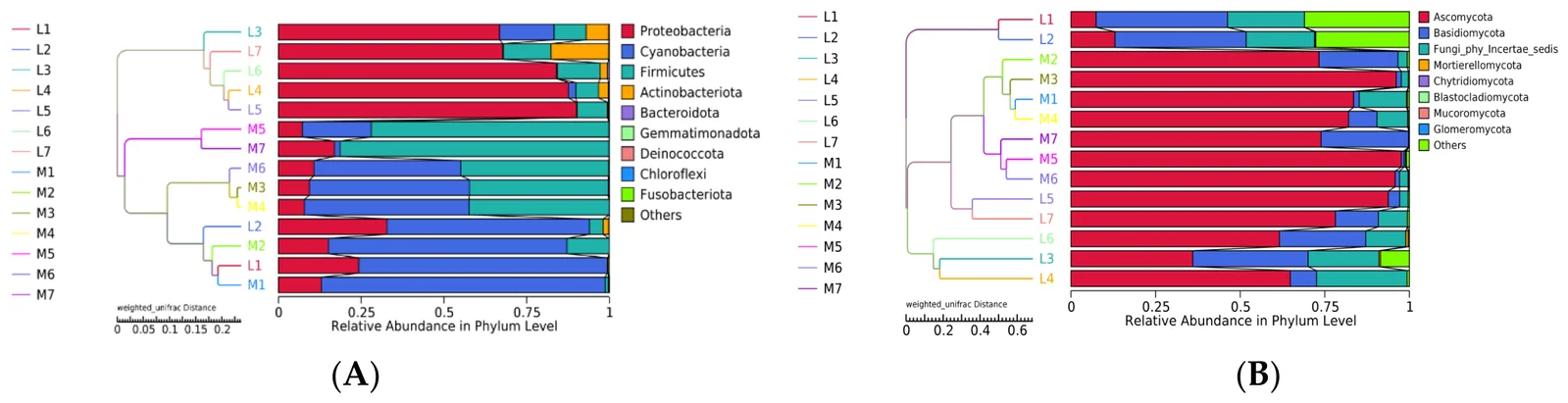
UPGMA cluster analysis of bacterial community (**A**) and fungal community (**B**) at different fermentation times in NFPP processed from GCP (L1–L7 represent 0, 1, 3, 7, 13, 15, 18 days) and BCP (M1–M7 represent 0, 1, 3, 7, 13, 15, 18 days).

A weighted Unifrac-based UPGMA clustering analysis was performed as shown in Figure 5A. Cluster I comprised L3, L4, L5, L6, and L7; Cluster II included M5 and M7; and Cluster III consisted of L1, L2, M1, M2, M3, M4, and M6. At a finer taxonomic level, ten samples—L3 and L7, L4 and L5, M5 and M7, M3 and M4, and L1 and M1—could be grouped into distinct subclusters, while the remaining four samples (L2, L6, M2, and M6) each formed their own separate branch.

The classification into these major clusters was primarily determined by the dominant bacterial phyla. Cluster I was characterized by samples where *Proteobacteria* was the most abundant phylum, followed by *Firmicutes*. The samples in Cluster II were predominantly dominated by *Firmicutes* with *Proteobacteria* as the secondary major phylum. In contrast, the classification of Cluster III was mainly attributed to *Cyanobacteria*, followed by *Proteobacteria* and *Firmicutes*. The clustering results reflect alterations in bacterial community structures commenced from the mid-fermentation stage in both GCP and BCP. As fermentation progressed, the bacterial community in GCP exhibited a developmental tendency towards *Proteobacteria*, whereas it shifted predominantly towards *Firmicutes* in BCP. These distinct directional shifts in the dominant phyla during fermentation are likely to impart different influences on the flavor profile of the final pickled pepper products.

As shown in Figure 5B, cluster I included L1 and L2, cluster II comprised M1–M7, L5 and L7, and cluster III consisted of L3, L4, and L6. Cluster I was characterized by a highest proportion of *Basidiomycota*, accompanied by only minor amounts of *Ascomycota*. Cluster II was defined by the predominance of *Ascomycota*, which accounted for over 75% of the fungal community in most samples. Cluster III was distinguished by a lower relative abundance of *Ascomycota* compared to Cluster II.

Fermentation samples in GCP were separated into three clusters, indicating continuous changes in its fungal community structure throughout the fermentation process. *Ascomycota* was the dominant phylum. In contrast, fermentation samples in BCP remained within a single major cluster, suggesting relative stability of its fungal community structure, which was consistently dominated by *Ascomycota*. By the late fermentation stage, the fungal community structures of GCP and BCP converged towards a certain degree of similarity.

#### 3.3.4. Cluster Analysis of Microbial Species Abundance

A cluster heatmap based on microbial species abundance was generated to clarify the similarities in bacterial and fungal community structures at the genus level.

**Figure 6:**
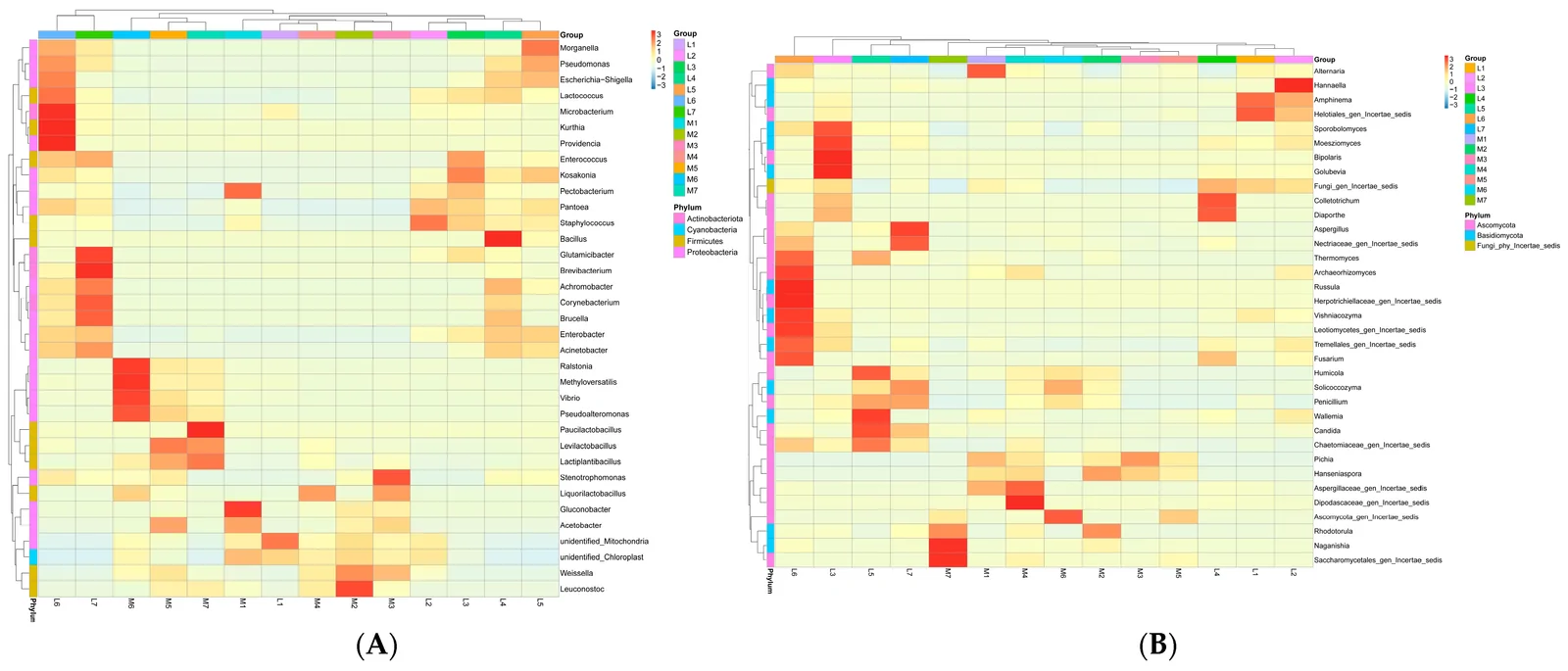
Top 35 clustering heat maps of horizontal species abundance of bacteria (**A**) and Fungi (**B**) at different fermentation times in NFPP processed from GCP (L1–L7 represent 0, 1, 3, 7, 13, 15, 18 days) and BCP (M1–M7 represent 0, 1, 3, 7, 13, 15, 18 days) (different colors represent different phyla as shown in the marked icons).

As shown in Figure 6A, hierarchical clustering of samples revealed four major phyla: *Actinobacteriota*, *Firmicutes*, *Proteobacteria*, and *Cyanobacteria*. The heatmap also revealed specific enrichments: *Pseudomonas* had the highest relative abundance in M2, *Gluconobacter* in M1, *Paucilactobacillus* in M7, while *Vibrio*, *Staphylococcus*, and *Brevibacterium* were most abundant in M6. *Achromobacter*, *Corynebacterium*, *Brucella*, *Enterobacter* and *Acinetobacter* were most abundant in L7. Among GCP samples, L2 and L3, L4 and L5, and L6 and L7 showed bacterial community similarity. Among BCP samples, M2 and M3, and M5 and M7 showed similarity. This confirms that bacterial community structure changes during fermentation and differs between pepper types.

In Figure 6B, hierarchical clustering of samples revealed three major phyla: *Ascomycota*, Basidiomycota and unidentified fungi. Specific enrichments included *Dipodascaceae* and *Aspergillaceae* being most abundant in M4; *Cephalosporium* and *Diaporthe* in L4; *Pichia* in M3; *Rhodotorula* and *Hanseniaspora* in M2; and *Moesziomyces* and *Bipolaris* in L3. During GCP fermentation, L1 and L2, L5 and L7 showed fungal community similarity. During BCP fermentation, M1 and M4, M3 and M5 showed similarity. This indicates that fungal community structure undergoes changes during fermentation and also differs between the two pepper types.

#### 3.3.5. Evolutionary Analysis of Microbial Communities at the Genus Level

To further investigate the phylogenetic relationships of the top microbial genera in the two pickled peppers, a phylogenetic tree was constructed using representative sequences from the top 100 genera obtained through multiple sequence alignment.

**Figure 7:**
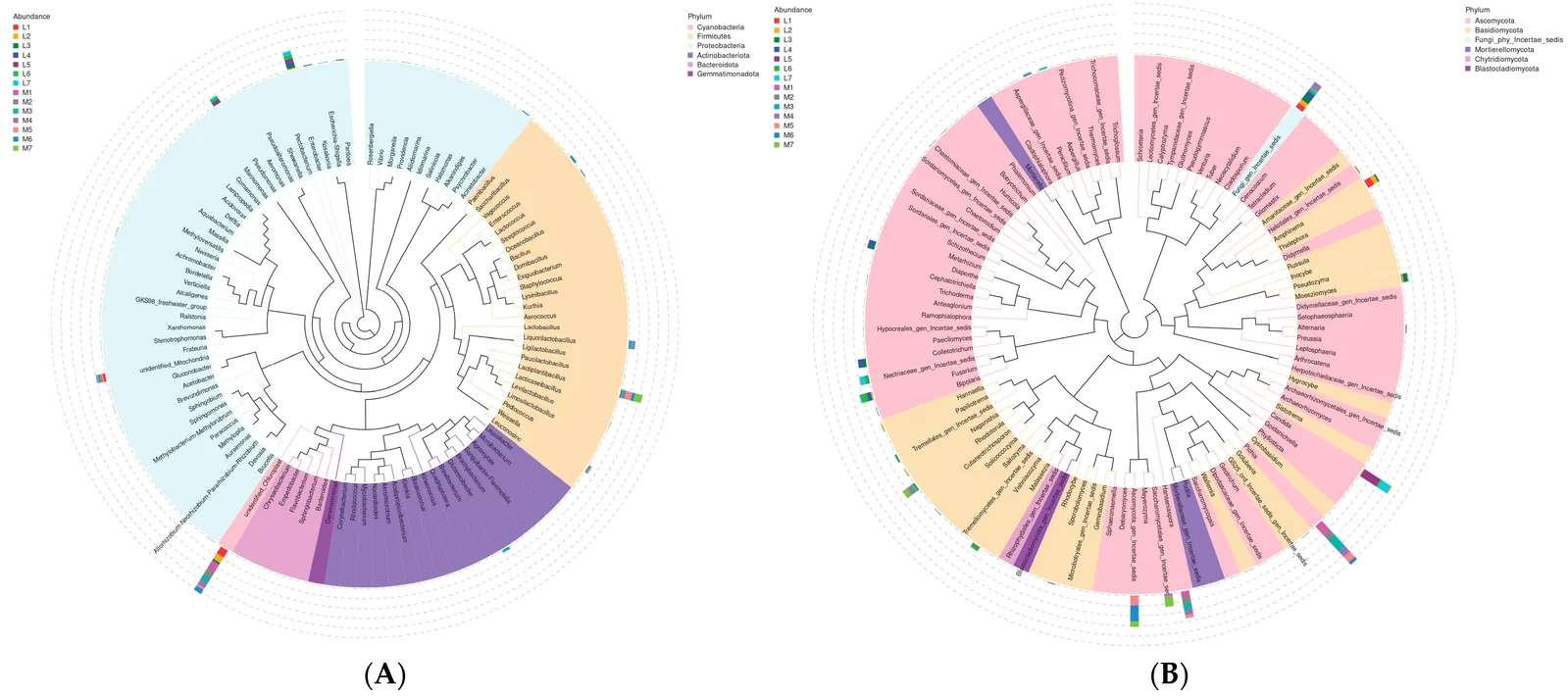
Horizontal species evolution tree of bacteria (**A**) and Fungi (**B**) at different fermentation times in NFPP processed from GCP (L1–L7 represent 0,1,3,7,13,15,18 days) and BCP (M1–M7 represent 0,1,3,7,13,15,18 days) (different colors represent different phyla as shown in the marked icons).

Figure 7A shows the bacterial phylogenetic tree. The top 100 bacterial genera were distributed across *Cyanobacteria*, *Firmicutes*, Proteobacteria, *Actinobacteriota*, *Bacteroidota*, and *Gemmatimonadota*, ranked by relative abundance. The highly abundant bacterial genera tended to be phylogenetically distant from each other.

Figure 7B shows the fungal phylogenetic tree. The top 100 fungal genera were distributed across *Ascomycota*, *Basidiomycota*, *Fungi phy Incertae sedis*, *Mortierellomycota*, *Chytridiomycota*, and *Blastocladiomycota*, ranked by relative abundance. Compared to bacteria, the dominant fungal genera were related more closely.

### 3.4. Correlation Analysis Between Microbe and Key Flavor Compounds

**Figure 8:**
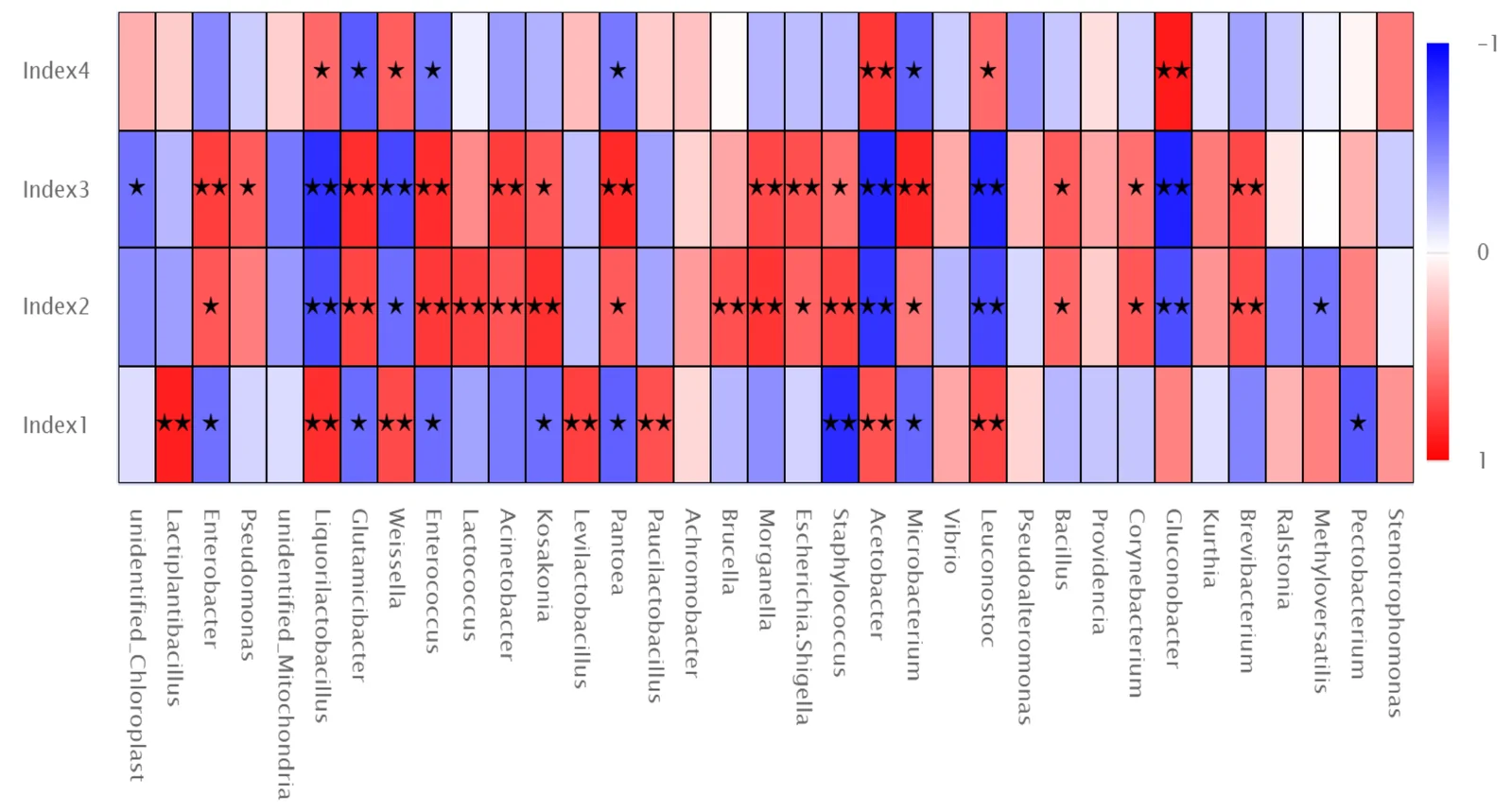
Correlation analysis of physical and chemical indexes between bacterial community and characteristic flavor substances in NFPP processed from GCP and BCP. (Index1–4 represent Lactic acid, Capsaicin, Volatile Flavor Substances and Free Amino Acid. “*” and “**” represent significance levels 0.05 and 0.01.

Lactic acid, free amino acids, volatile flavor compounds, and capsaicin are the four major components that constitute the characteristic flavor of naturally fermented pickled peppers. Changes in the content of these 4 major components will lead to changes in the characteristic flavor. A correlation analysis was conducted between the microbes and four indexes: lactic acid (Index1), capsaicin (Index2), volatile flavor compounds (Index3), and free amino acids (Index4). The results are shown in Figure 8. In the figure, * and ** indicate significance levels of 0.05 and 0.01, respectively [21].

#### 3.4.1. Correlation Analysis Between Bacteria and Key Flavor Compounds

As shown in Figure 8, among the top 35 bacteria, there were 17 bacterial species showing a highly significant positive correlation (*p* ≤ 0.01) with the four major flavor components, 4 species showing a highly significant negative correlation (*p* ≤ 0.01), and 1 species showing different correlations with different flavor compounds. There were seven bacterial species showing a significant positive correlation (*p* ≤ 0.05), four species showing a significant negative correlation (*p* ≤ 0.05), and three species showing different correlations with different flavor compounds.

The correlation analysis with flavor compounds revealed that the correlation patterns of bacteria with capsaicin and volatile flavor compounds were similar. This may be due to a selective effect of capsaicin on bacterial succession, and the thriving bacteria likely produced similar metabolites, thereby forming analogous volatile flavor compounds. Lactic acid is a characteristic flavor compound in pickled peppers. Bacteria strongly correlated with lactic acid showed weak or negative correlations with the first two types of compounds, while bacteria strongly correlated with free amino acids showed weak or negative correlations with the other three types of compounds [22].

#### 3.4.2. Correlation Analysis Between Fungi and Key Flavor Compounds

**Figure 9:**
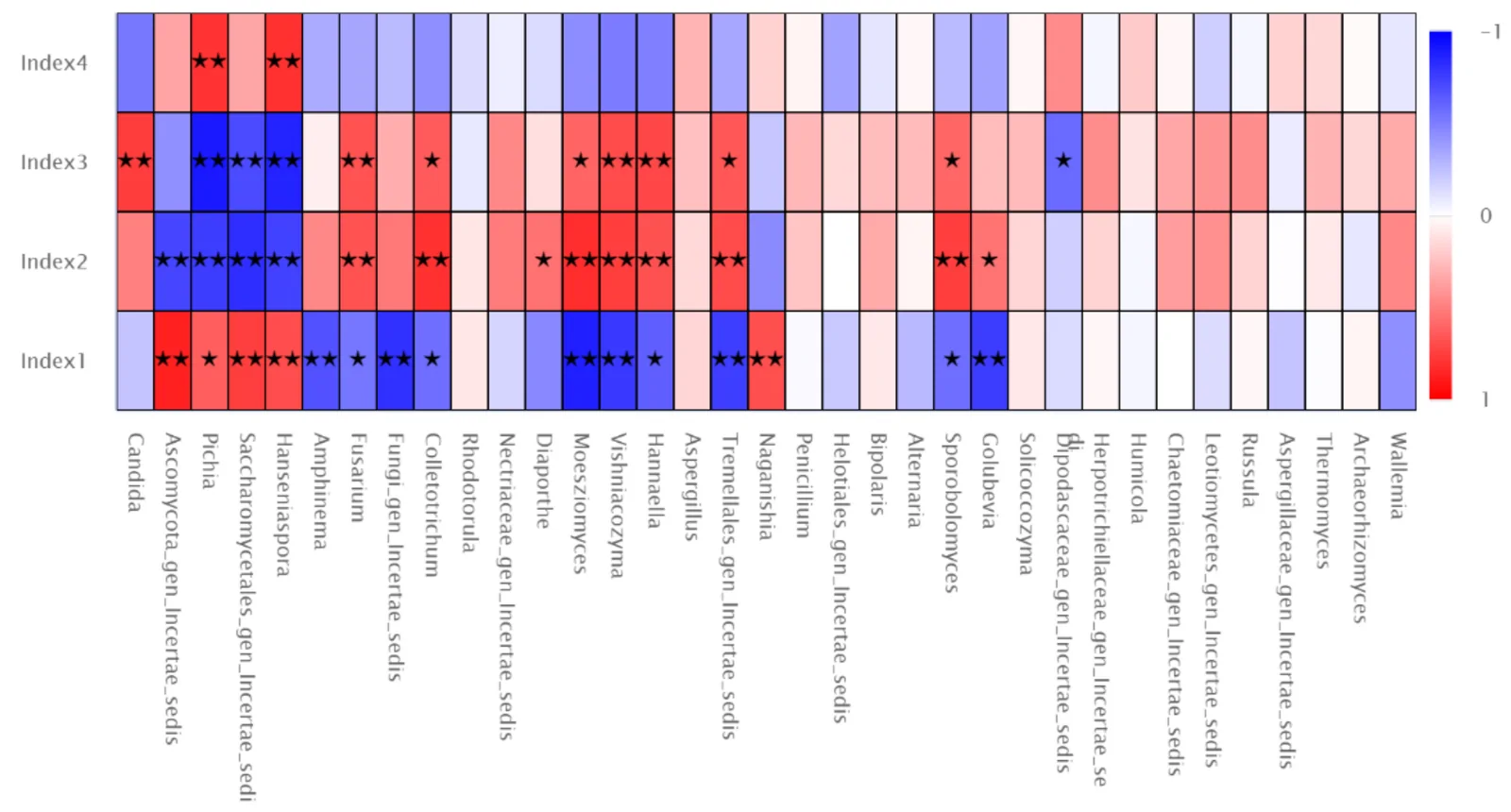
Correlation analysis of physicochemical indexes between fungal community and characteristic flavor substances in NFPP processed from GCP and BCP. (Index1–4 represent Lactic acid, Capsaicin, Volatile Flavor Substances and Free Amino Acid. “*” and “**” represent significance level 0.05 and 0.01).

Fungi are important microorganisms influencing the flavor of pickled peppers in the later stages of fermentation and probably contribute to the flavor through ester formation. As shown in Figure 9, there were four fungal species showing a highly significant positive correlation (*p* ≤ 0.01) with the four major flavor components, one species showing a highly significant negative correlation (*p* ≤ 0.01), and six species showing different correlations with different flavor compounds. There were three fungal species showing a significant positive correlation (*p* ≤ 0.05), two species showing a significant negative correlation (*p* ≤ 0.05), and two species showing different correlations with different flavor compounds.

The correlation analysis with flavor compounds found that fungi may exhibit correlation patterns similar to those of bacteria. More diverse fungi in the later stages of fermentation may lead to a greater variety of flavor compounds—[23,24].

#### 3.4.3. Variation of Flavor-Related Microbes During the Natural Fermentation of GCP and BCP

Variation of microbial communities in relatively high abundance during different fermentation periods is shown in Table 1 and Table 2.

During the fermentation of GCP, although the bacteria-associated flavor compounds show some fluctuations, they all maintain a relatively high relative abundance in the mid-to-late fermentation stages (after 7 days). This indicates that these flavor-associated bacteria maybe play a major role in the mid-to-late stages. Among fungi, only the *genus Candida* exhibits a high relative abundance in the later stage (after 15 days), while other fungi dominate in the early fermentation stage.

During the fermentation of BCP, the flavor-associated bacteria *Weissella*, *Lactococcus*, and *Pantoea* are active in the early stage, while other bacteria are active in the mid-to-late stages. Among the flavor-associated fungi, *Candida*, *Pichia*, and *Hanseniaspora* are active in the early stage, and other fungi are active in the mid-to-late stages [23].

## 4. Conclusions

Analysis of the microbial community structure at the genus level revealed that the dominant bacterial genera (with relative abundance exceeding 0.01%) were eight in GCP and seven in BCP; *Lactiplantibacillus*, *Weissella*, *Lactococcus* and *Pantoea* were the same dominant genera in BCP and GCP. In the fungal genus level, the dominant taxa (relative abundance > 0.01%) consist of seven in GCP and five in BCP; *Candida*, *Rhodotorula* and *Fusarium* existed in both GCP and BCP.

Taxonomic composition analysis in the genus level revealed bacterial communities exhibited successional variation, while fungal communities showed no clear successional pattern. In the early fermentation stage (days 0–7) of GCP were *Weissella* and *Pantoea, followed by Enterobacter*, *Pseudomonas* and *Glutamicibacter* in the mid to late fermentation stages (days 8–18), while in BCP, the dominant bacteria were *Liquorilactobacillus* and *Weissella* in the early stage (days 0–7), and *Lactiplantibacillus* emerged as the predominant genus in the mid to late fermentation stages (days 8–18) [25].

*Lactobacillus* and *Candida* were the same microbes associated with their characteristic flavor in both GCP and BCP. Other microbes such as *Acinetobacter* in GCP and *Weissella* in BCP contribute unique flavor. As GCP and BCP are different in pepper type and ripeness, this may lead to a little uncertainty in the conclusion, but there are differences in microbial species associated with characteristic flavor in GCP and BCP [24].

## Figures and Tables

**Table 1:** Variation of relative abundance of bacteria- and fungi- related (in genus level) characteristic flavor substances at different fermentation time in NFPP processed from GCP.

Microbe	Genus	Relative Abundance (%)						
		0 d	1 d	3 d	7 d	13 d	15 d	18 d
bacteria	*Lactobacillus*	0.12	0.25	0.32	2.74	5.44	3.12	6.58
	*Enterococcus*	0.20	10.71	21.69	35.18	33.62	32.63	36.02
	*Pseudomonas*	0.03	0.03	0.23	14.31	23.17	25.67	12.11
	*Glutamicibacter*	0.01	1.61	6.60	2.41	0.17	0.66	16.09
	*Lactococcus*	0.21	1.04	1.65	1.93	0.80	3.22	0.84
	*Acinetobacter*	0.10	0.05	0.45	2.24	1.85	2.23	3.11
	*Pantoea*	0.12	2.55	2.15	1.23	1.91	2.26	1.53
	*Brucella*	0	0.01	0.01	0.81	0.02	0.49	1.31
	*staphylococcus*	0.04	1.08	0.70	0.43	0.44	0.20	0.27
	*Microbacterium*	0.20	0.09	0.12	0.08	0.05	0.91	0.17
fungi	*Candida*	0.63	2.76	1.19	1.43	83.68	7.75	46.21
	*Dipodichlamys*	29.77	21.87	6.36	0.50	0.05	0.01	0
	*Fusarium*	0.83	5.45	1.11	17.29	1.05	28.70	2.16
	*Moesziomyces*	2.40	5.09	14.79	3.43	0.46	1.27	2.95

**Table 2:** Variation of relative abundance of bacteria- and fungi-related (in genus level) characteristic flavor substances at different fermentation time in NFPP processed from BCP.

Microbe	Genus	Relative Abundance (%)						
		0 d	1 d	3 d	7 d	13 d	15 d	18 d
bacteria	*Lactobacillus*	0.02	1.36	13.02	16.29	59.98	26.45	73.68
	*Enterococcus*	0.38	0.10	0.09	0.01	0.16	0.03	3.92
	*Lactobacillus glycogenica*	0	0.24	20.26	20.20	2.77	14.90	0.82
	*Weissella*	0.50	9.92	7.92	4.73	5.60	3.04	1.21
	*Lactococcus*	0.04	0.31	0.19	0.05	0.14	0.04	0.01
	*Pantoea*	0.68	0.07	0.03	0.01	0.10	0.04	0.40
	*Liquorilactobacillus*	0.01	0.10	0.11	0.48	3.06	0.04	2.77
	*Acetobacter*	1.03	0.43	0.76	0.08	1.03	0.08	0.05
fungi	*Candida*	0.04	0.01	0.02	3.00	0.02	0.16	4.49
	*Ascomycota_gen_incerta_sedis*	0.10	0.04	0.14	3.00	40.40	72.79	25.29
	*Pichia*	54.65	33.33	66.63	36.59	32.91	13.98	1.54
	*Candida hansenii species*	23.05	35.51	26.11	24.43	14.59	0.39	0.35
	*fusarium*	0.25	0.57	0.61	0.39	0.68	1.12	0.02

## Data Availability

More data is available by request or on the website: https://magic.novogene.com/ under the Project X101SC23101687-Z01.

## Appendix A

**Table A1:** Free Amino Acids (FAA) of GCP at different fermentation times.

Flavor	FAA	Content mg/Kg						
		0 d	1 d	3 d	7 d	13 d	15 d	18 d
Umani	Asp.	2.5	135.1	115.9	89.3	109.1	298.7	497.7
	Glu.	15.5	121.5	167.9	305.1	116.9	155.8	165.6
Sweet	Thr.	51.1	52.0	83.8	142.3	136.7	130.3	132.1
	Ala.	80.9	233.5	223.2	213.1	376.8	356.1	287.2
	Ser.	394.1	423.5	294.9	483.2	660.5	681.4	642.4
	Gly.	155.9	303.4	332.1	251.5	407.5	450.1	489.2
Bitter	His.	498.5	226.3	242.8	162.4	120.5	116.1	157.2
	Arg.	43.2	160.1	182.3	175.2	153.7	127.2	167.3
	Val.	25.5	97.9	60.3	163.1	116.3	176.1	109.5
	Lys.	64.2	90.6	81.8	95.1	87.4	74.3	69.1
	Pro.	14.2	163.6	175.1	419.3	303.2	304.5	176.4
	Ile.	10.1	107.7	137.5	122.8	83.6	73.9	148.1
	Leu.	63.7	112.3	163.2	137.8	88.6	91.4	97.6
	Phe.	50.1	83.1	67.9	90.2	67.8	94.1	88.4
Non-taste	Tyr.	21.5	59.7	75.5	57.8	44.4	41.4	42.1
	Cys.	28.6	79.2	83.8	76.7	58.9	55.1	55.8
Total		1519.6	2449.5	2488.0	2984.9	2931.9	3226.5	3325.7

**Table A2:** Free Amino Acids(FAA) of BCP at different fermentation times.

Flavor	FAA	Content mg/Kg						
		0 d	1 d	3 d	7 d	13 d	15 d	18 d
Umani	Asp.	629.9	539.1	213.5	185.6	159.3	146.2	87.1
	Glu.	459.6	103.7	78.8	125.8	214.9	78.1	93.6
Sweet	Thr.	446.7	425.4	183.1	245.5	183.9	281.3	57.6
	Ala.	655.9	671.1	359.9	154.1	265.5	437.3	191.7
	Ser.	1615.8	1373.3	992.6	796.1	554.6	967.7	571.2
	Gly.	760.3	699.6	395.8	275.2	257.1	521.7	313.1
Bitter	His.	259.7	290.1	159.8	204.7	193.1	24.1	38.3
	Arg.	409.1	591.3	270.7	121.8	331.6	72.8	99.1
	Val.	438.8	366.1	175.7	154.4	164.6	174.3	113.5
	Lys.	450.2	402.1	164.6	124.1	132.9	126.7	159.1
	Pro.	603.3	436.8	330.1	606.7	250.4	116.6	150.5
	Ile.	415.6	307.5	117.8	74.8	158.8	83.9	42.7
	Leu.	473.4	420.5	135.2	190.1	130.1	111.2	121.9
	Phe.	453.3	335.6	107.8	119.9	174.5	78.2	76.9
Non-taste	Tyr.	389.2	345.7	44.8	60.1	124.8	56.3	38.1
	Cys.	516.2	458.4	59.4	79.6	165.5	74.7	50.3
Total		8977.0	7766.3	3789.6	3518.5	3461.6	3351.1	2204.7
